# Real time organ hypoperfusion detection using Indocyanine Green in a piglet model

**DOI:** 10.1007/s00464-024-10938-0

**Published:** 2024-06-13

**Authors:** Carolin Oppermann, Niclas Dohrn, Helin Yikilmaz Pardes, Mads Falk Klein, Thomas Eriksen, Ismail Gögenur

**Affiliations:** 1 Center for Surgical Science, Zealand University Hospital, Lykkebækvej 1, 4600 Køge, Denmark; 2 Department of Surgery, Copenhagen University Hospital, Herlev & Gentofte, Borgmester Ib Juuls Vej 1, 2730 Herlev, Denmark; 3 Institute for Clinical Veterinary Medicine, University of Copenhagen, Dyrelægevej 16, 1870 Frederiksberg C, Denmark

**Keywords:** Indocyanine Green, Hypoperfusion, Fluorescence-angiography, Fluorescence, Abdominal surgery, Porcine study

## Abstract

**Background:**

Preserving sufficient oxygen supply to the tissue is fundamental for maintaining organ function. However, our ability to identify those at risk and promptly recognize tissue hypoperfusion during abdominal surgery is limited. To address this problem, we aimed to develop a new method of perfusion monitoring that can be used during surgical procedures and aid surgeons’ decision-making.

**Methods:**

In this experimental porcine study, thirteen subjects were randomly assigned one organ of interest [stomach (*n *= 3), ascending colon (*n *= 3), rectum (*n *= 3), and spleen (*n *= 3)]. After baseline perfusion recordings, using high-frequency, low-dose bolus injections with weight-adjusted (0.008 mg/kg) ICG, organ-supplying arteries were manually and completely occluded leading to hypoperfusion of the target organ. Continuous organ perfusion monitoring was performed throughout the experimental conditions.

**Results:**

After manual occlusion of pre-selected organ-supplying arteries, occlusion of the peripheral arterial supply translated in an immediate decrease in oscillation signal in most organs (3/3 ventricle, 3/3 ascending colon, 3/3 rectum, 2/3 spleen). Occlusion of the central arterial supply resulted in a further decrease or complete disappearance of the oscillation curves in the ventricle (3/3), ascending colon (3/3), rectum (3/3), and spleen (1/3).

**Conclusion:**

Continuous organ-perfusion monitoring using a high-frequency, low-dose ICG bolus regimen can detect organ hypoperfusion in real-time.

4.2 million people are estimated to die from perioperative complications each year [1, 2]. Factors impacting perioperative death can be numerous, and undetected hypoperfusion to vital organs is one of them. Preserving sufficient oxygen supply to the tissue is fundamental in maintaining organ function [3, 4]. This is particularly relevant when metabolic needs change markedly, for example, in response to major surgery or critical illness [5, 6].

However, our ability to identify those at risk and promptly recognize tissue hypoperfusion is limited during surgery, and especially in abdominal surgery, vascular anatomy varies [7]. During colorectal resections, for instance, the blood supply to the organ is embedded and hidden away in the mesentery and requires skilled surgical experience to achieve proper target resection without damaging blood supply to healthy tissue [8]. For example, collateral supply to the remnant colon after high-tie ligation of the inferior mesenteric artery in left-sided colectomies or rectal resections is not always sufficient, increasing the risk for anastomotic leakage, organ dysfunction, and mortality [9].

Indocyanine Green (ICG) is a fluorophore used in many medical specialties, including abdominal surgery, where it helps assess organ blood perfusion. To evaluate perfusion, ICG is currently given as a single-dose intravenous bolus injection [10].

When exposed to near-infrared light (NIR), fluorescence from the ICG particles is observed. This signal can be evaluated in two ways: (1) Qualitatively, by subjectively looking at the fluorescent signal, or (2) quantitatively, by computational pixel analysis to provide the surgeon with unbiased information on the perfusion status [11–14]. The ICG bolus is given intraoperatively and due to its half-life of 3–5 min allows for only a transient evaluation of the blood perfusion before it is cleared from the bloodstream and excreted into the bile by the liver.

Our research group has recently published a new method for continuous perfusion monitoring using a high-frequency, low-dose ICG bolus regimen that repetitively produces small ICG signals too small to be assessed subjectively [15].

In this study, we wanted to investigate whether hypoperfusion generated through occlusion of the arterial supply could be detected in a setting where continuous perfusion monitoring was performed.

## Methods

### Animals

The study was conducted at the University of Copenhagen’s Department of Experimental Medicine (AEM). In this non-survival investigation, thirteen female domestic pigs weighing between 16 and 32 kg were sacrificed. We included only pigs that were clinically healthy, free of disease, and with a normal body temperature. If an animal died or had to be euthanized early during surgery due to hemodynamic instability, it was removed from the study.

The research was carried out under the supervision and approval of the regional animal ethics committee (Approval Nr. 2021–15-0201–00924) and in compliance with the ARRIVE guidelines [16].

#### Anesthesia, fluid administration, ventilation, and euthanasia

Before surgery, the animals were sedated with i.m. 20-25mg/kg ketamine and i.m. 0.5–0.7 mg/kg midazolam. Ear veins were cannulated bilaterally, and if necessary, anesthesia was deepened with 1–4 mg/kg propofol before oro-tracheal intubation. Anesthesia was maintained with sevoflurane 2–2.5%. All pigs were mechanically ventilated, and settings were aimed at normoventilation and FiO2 at 60%.

Analgesia was achieved with a bolus of 5–20 µg/kg fentanyl i.v. and followed with a continuous rate infusion of 10–100 µg/kg/h fentanyl i.v. depending on the response.

The femoral artery and vein were cannulated for fluids and ICG administration. Vital parameters, i.e., ETCO2, SpO2, ECG, and arterial blood pressure, were continuously monitored. At the end of the surgery, the anesthetized animals were euthanized by a rapid i.v. injection of 40 mmol potassium chloride. ECG and blood pressure recordings confirmed asystole and circulatory arrest.

#### Surgical preparations and measurements

Four abdominal organs; the stomach, ascending colon, rectum, and spleen, were randomly assigned to each of the twelve study subjects. Three laparoscopic ports (5–12 mm) were placed inside the abdominal cavity after CO2 insufflation to 12 mmHg. We visualized the organ of interest using a laparoscopic camera (Stryker, 1588 AIM HD Camera System) and mounted the camera in a stationary mechanical arm at a 7.5 cm distance from the organ of interest.

After administering a priming dose, we initiated the high-frequency (1 per min) low-dose bolus administration of ICG using an infusion pump (Micrel RythmicTM Evolution) as described previously.

Measurement of all four target organs started from a 7.5 cm distance with a baseline measurement for 6 min. Hypoperfusion was induced via occlusion of selected arteries, moving from peripheral to central arteries to simulate various degrees of hypoperfusion (mild and severe hypoperfusion) using electrical ligation and division of arteries (Covidien LigaSure™ Laparoscopic Sealer/Divider). Occlusion of the peripheral arterial branches was followed by a 6-min measurement without any further intervention. The camera distance was then increased to 15 cm, followed by a 6-min measurement. Subsequently, a central artery was occluded, and the recording continued for another 6 min at 7.5 and 15 cm distances.

We identified the arteries most suitable for occlusion in the respective organs [17, 18]. In the stomach, we occluded the left and subsequent right gastroepiploic arteries to produce mild and severe hypoperfusion of the major curvature of the ventricle. To produce mild and subsequent severe hypoperfusion in the ascending colon, we occluded the ileocolic and cranial mesenteric artery. Hypoperfusion in the rectum was caused by occluding the caudal mesenteric and right colic artery. The spleen was deprived of its arterial blood supply by occluding the splenic and left gastroepiploic artery.

If collateral perfusion counteracted the development of severe hypoperfusion or ischemia, then the cranial mesenteric artery and the proximal hepatic or coeliac artery were occluded. Occlusion of vessels was permanent.

#### Method of quantitative perfusion assessment

*PerfusionWorks* (PerfusionTech Aps, Denmark) is an image-analyzing software developed for real-time, intraoperative quantitative ICG assessment. The software is operated from an external tablet or laptop with video input from the surgical platform, but it can also be used to analyze existing video material. In theory, an intravenously administered bolus of ICG can be seen moving through the vascular system in a single wave-like manner. Repeated bolus administration in perfused tissue, therefore, leads to continuous waves that can be recorded as oscillation curves. Subsequently, damage to the arterial supply will result in a change (e.g., flattening) of the oscillation curve recording—visualizing poor perfusion.

The software allows the surgeon to choose size-adjustable regions of interest (ROI), where the oscillation curves are recorded and analyzed. From each ROI, the software calculates various perfusion metrics and creates time-intensity curves that can give information on the perfusion status of the specific anatomical area. In this experimental study, we selected a central and peripheral ROI for perfusion quantification analysis during each condition (baseline, mild, and severe hypoperfusion).

**Fig. 1:**
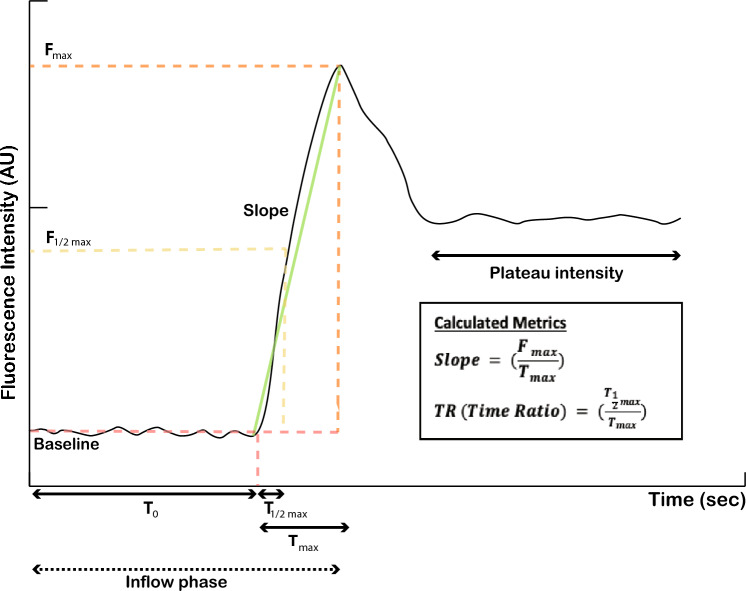
A graphic depicting different quantitative ICG metrics, including ${{\varvec{F}}}_{{\varvec{m}}{\varvec{a}}{\varvec{x}}}$—maximal fluorescent signal intensity, ${{\varvec{T}}}_{0}$—time from ICG injection till first recording of fluorescent signal, ${{\varvec{T}}}_{{\varvec{m}}{\varvec{a}}{\varvec{x}}}$—time from first fluorescent signal to maximal intensity, and—${{\varvec{T}}}_{\frac{1}{2}{\varvec{m}}{\varvec{a}}{\varvec{x}}}$time until 50% of maximal fluorescence intensity is reached

The software’s tracking algorithm mitigates the impact of the intestines’ movement during the respiratory cycle and intestinal peristalsis. The result is an ICG fluorescence time-intensity curve from which the perfusion metrics are extracted. The following perfusion metrics were analyzed in this study: ***Slope***, **F**_max_, **T**_0,_ **T**_max_, **T**_1/2 max,_ and **TR** (Fig. 1). Means and standard deviations (SD) were calculated based on all time-intensity curve metrics in each condition.

## Results

Twelve out of thirteen subjects completed the experiment. During the preparation of the arterial supply of the spleen, one pig suffered from unmanageable bleeding from the splenic artery and was therefore euthanized prematurely. All other animals remained hemodynamically stable.

To quantitatively assess organ perfusion, fluorescence intensity and perfusion time metrics were calculated per ICG bolus from each ICG fluorescence graph.

**Fig. 2:**
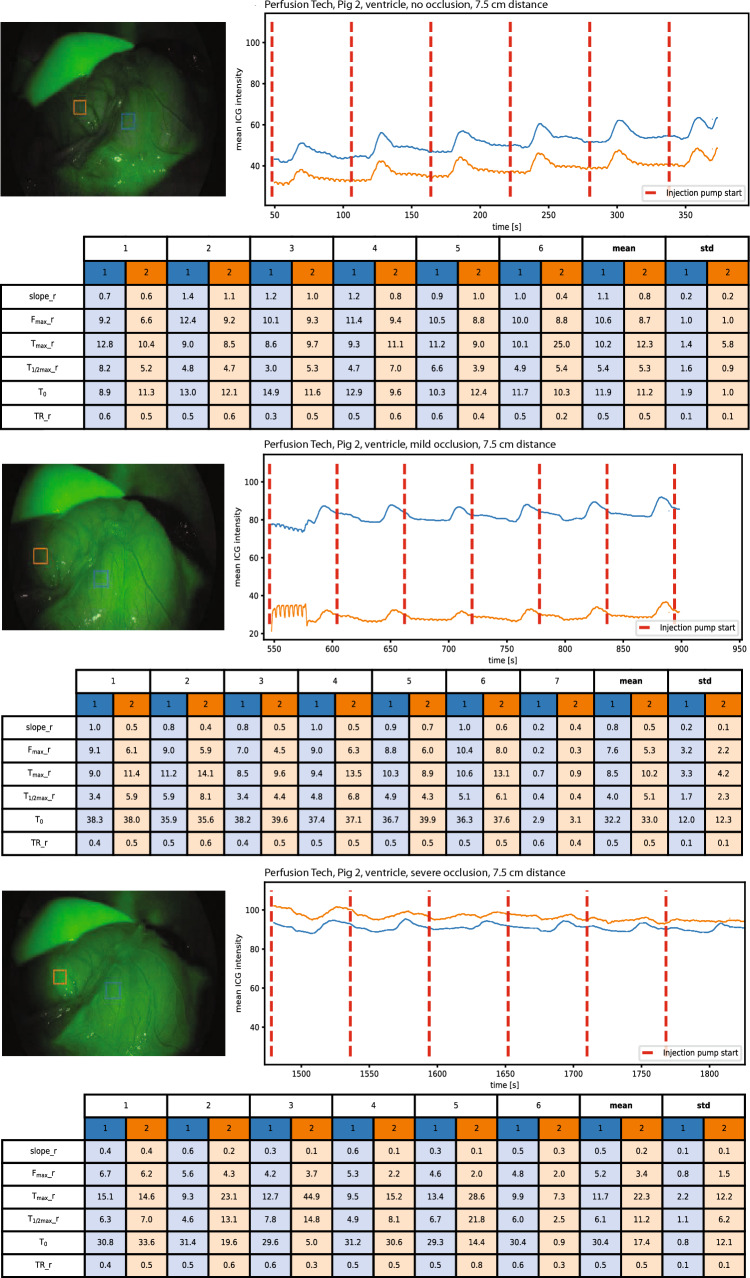
An example of baseline perfusion, mild and severe hypoperfusion in the ventricle with perfusion metrics. Blue curve: Region of interest 1 (ROI central), Orange curve: Region of interest 2 (ROI peripheral) (Color figure online)

**Fig. 3:**
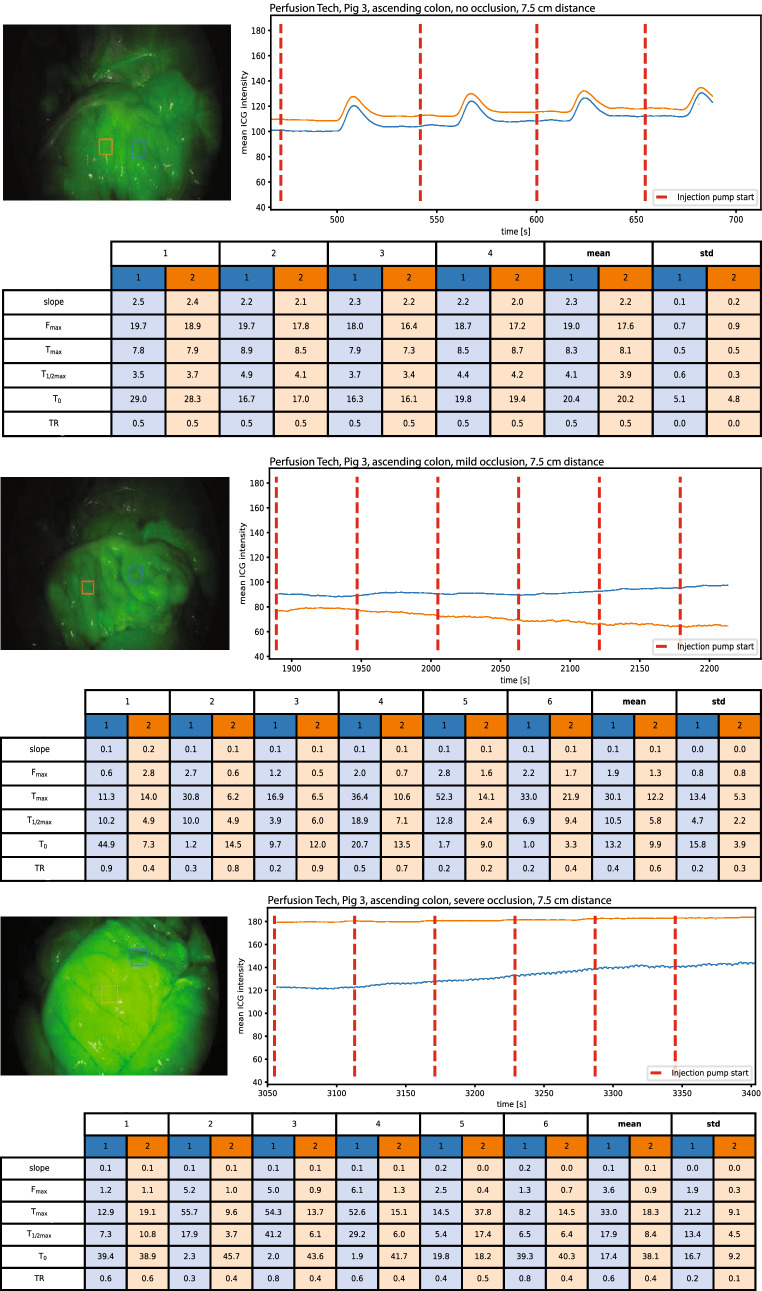
An example of baseline perfusion, mild and severe hypoperfusion in the ascending colon with perfusion metrics. Blue curve: Region of interest 1 (ROI central), Orange curve: Region of interest 2 (ROI peripheral) (Color figure online)

**Fig. 4:**
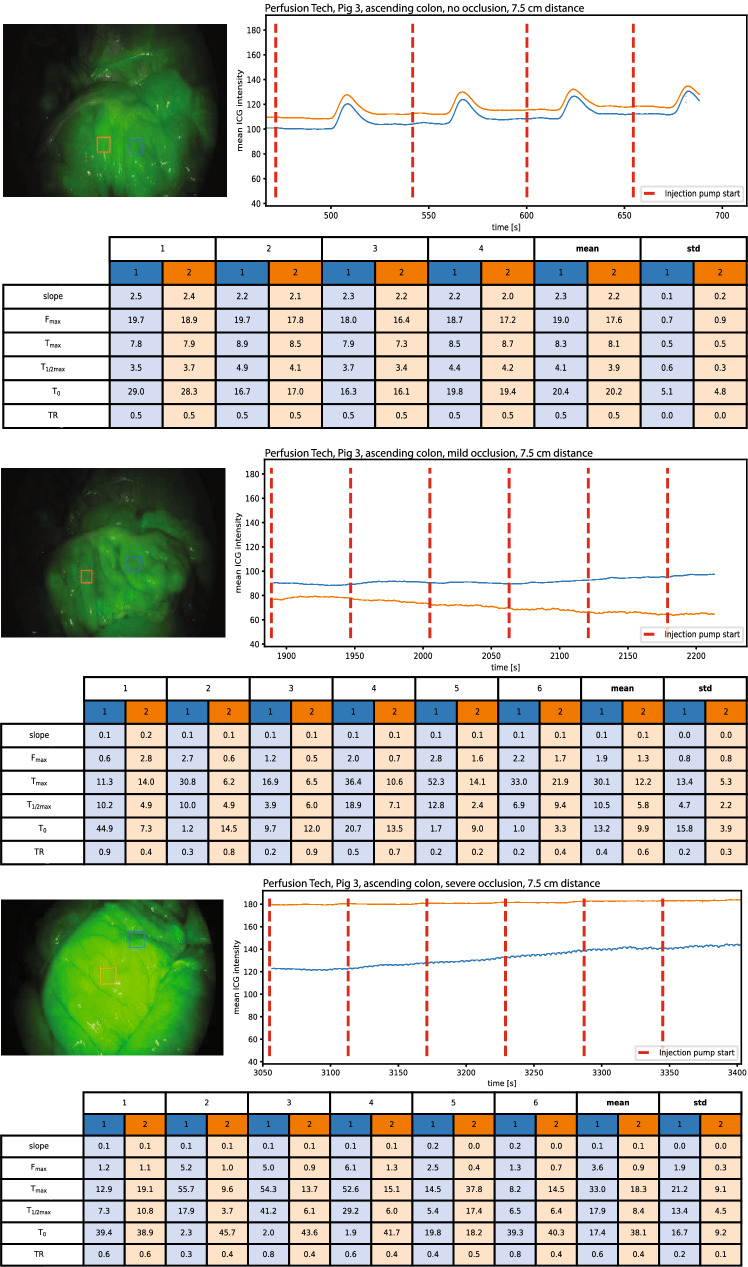
An example of baseline perfusion, mild and severe hypoperfusion in the rectum with perfusion metrics. Blue curve: Region of interest 1 (ROI central), Orange curve: Region of interest 2 (ROI peripheral) (Color figure online)

**Fig. 5:**
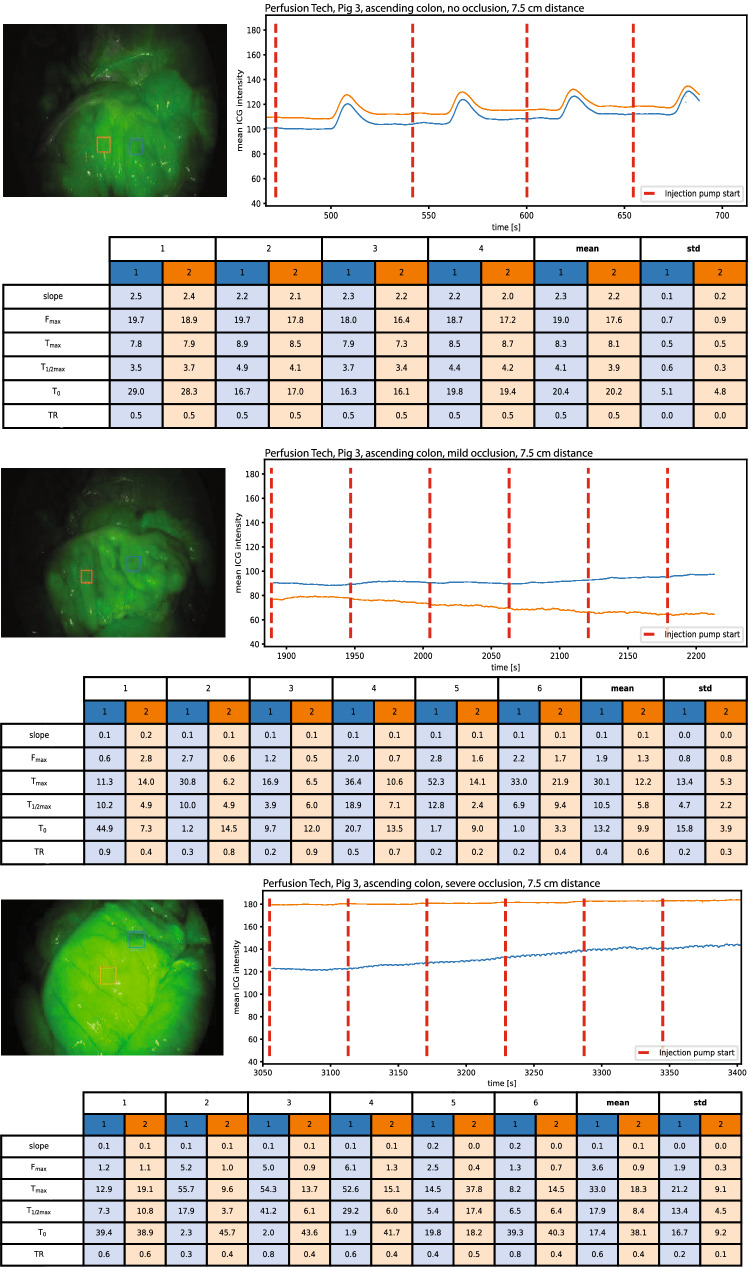
An example of baseline perfusion, mild and severe hypoperfusion in the spleen with perfusion metrics. Blue curve: Region of interest 1 (ROI central), Orange curve: Region of interest 2 (ROI peripheral) (Color figure online)

Baseline ICG signal was recorded in the ventricle (3/3) (Fig. 2), ascending colon (3/3) (Fig. 3), rectum (3/3) (Fig. 4), and in the spleen (2/3) (Fig. 5).

Occlusion of the peripheral arterial supply (mild hypoperfusion) translated in an immediate decrease in oscillation signal in most organs (3/3 ventricle, 3/3 ascending colon, and 3/3 rectum), while we were only able to detect small changes in the perfusion status in two spleens (2/3).

Occlusion of the central arterial supply (severe hypoperfusion) resulted in a further decrease or complete disappearance of the oscillation curves in the quantitative ICG-signal in the ventricle (3/3), ascending colon (3/3), rectum (3/3), and spleen (1/3).

**Fig. 6:**
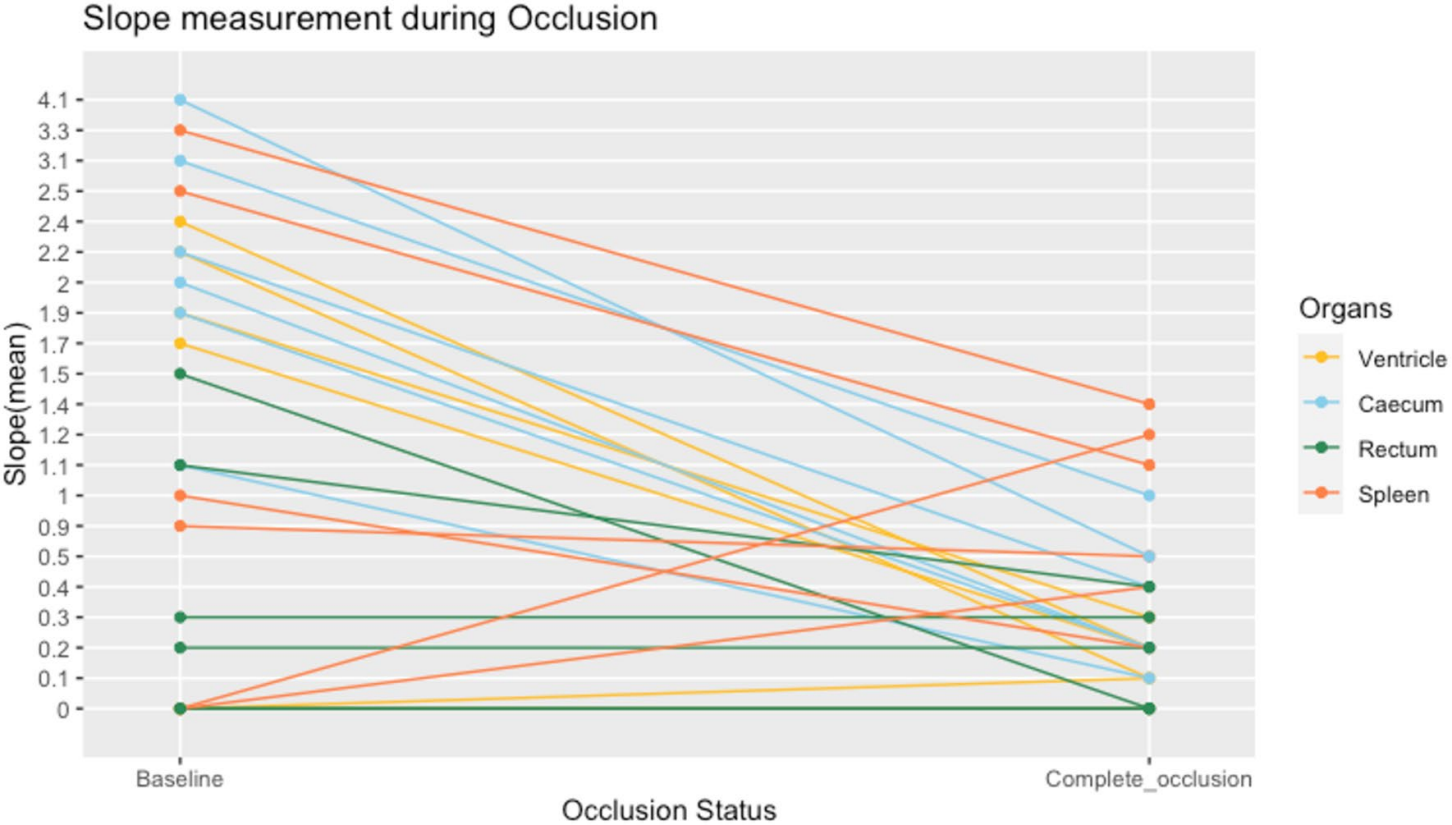
A graphic depiction of Slope as measured during baseline and after arterial occlusion

Slope notably decreased after the arterial supply was occluded, moving toward zero in most organs (2/3 ventricle, 3/3 caecum, 1/3 rectum, and 2/3 spleen) (Fig. 6).

Furthermore, a paired t-test showed that mean Slope significantly decrease when comparing baseline measurements (M = 1.39, SD = 1.24) to complete occlusion measurements (M = 0.39, SD = 0.41), (*P *< 0.001).

During the experiment, we observed that the initial priming dose did not lead to a clear fluorescent signal in each organ. We decided to adhere to the original trial protocol and did not adjust the priming dose during the trial run.

Furthermore, we observed a visual saturation of ICG signal, making qualitative assessment of the perfusion status, including assessment of individual oscillation curves, impossible. However, the quantification software was able to detect hypoperfusion in brightly fluorescent organs.

## Discussion

This experimental study shows that continuous organ-perfusion monitoring with ICG can assess changes in organ perfusion and adequately detect hypoperfusion in real time.

After inducing mild and severe hypoperfusion in the ventricle, ascending colon, rectum, and spleen, the effect on the recorded oscillation curves, compared to the previously recorded baseline perfusion, was visible almost immediately. Our results show that hypoperfusion resulted in either a lower curve amplitude, flattening or complete disappearance of the oscillation curves—indicating poor or absent perfusion to the target ROIs.

This was well reflected by ***Slope*** significantly decreased after the surgical intervention. As of today, no consensus has been reached about which perfusion metrics hold the most value in perfusion assessment, with some studies suggesting **T**_**0**_ to be the strongest indicator of hypoperfusion, while other research points to ***Slope*** being of high significance during perfusion monitoring [12–14, 19]. In an RCT including 68 patients, ***Slope*** furthermore appeared to mirror differences in the manipulation of the intestines accurately [20]. Our results strengthen the assumption that ***Slope*** seems most suitable to reflect changes in perfusion status, decreasing in most organs as soon as hypoperfusion was induced.

Four organ measurements show an increase in Slope after occlusion. This is likely due to insufficient priming of the organ before the baseline measurement.

While executing this study, we discovered that the pre-defined ICG priming dose was insufficient to achieve a clear perfusion signal recording in all organs. While measurement of Slope, as well as other metrics, was achieved in two/third of all measurements, the priming dose was too small to result in a proper ICG signal in one ventricle, two rectum, and one spleen measurements. During the experiment, our team decided to adhere to the protocol. However, looking at our results, we will, from now on, advocate that each organ requires an individual priming dose to achieve a good enough perfusion signal for monitoring. Furthermore, collateral arteries might have supplied the organ even after the surgical intervention.

A qualitative organ assessment of the ICG signal would have been misleading. Due to continuous bolus administration over the surgical course, the ICG concentration had led to visual saturation of the target organs, reflected by an increasing mean ICG intensity after each bolus in all organ recordings. Despite quantitatively well-documented hypoperfusion, bright fluorescence could be observed, making qualitative evaluation impossible or fallible.

Current perfusion monitoring during surgery is predominately done by measuring cardiac output, oxygen delivery, and oxygen consumption [21]. While these measurements provide general information on the patient’s hemodynamic status, they fail to address possible complications at the organ level.

To give an example, abdominal surgery often necessitates extensive surgical dissection of the mesentery, mobilization of organs, and ligation of vessels with varying anatomy. Limited oversight or clinical suspicion of hypoperfusion can make it difficult to detect it at occurrence.

To our knowledge, this is the first time continuous perfusion monitoring with ICG has been used to observe and detect organ hypoperfusion in real-time during surgery, addressing the abovementioned problem with a new, promising monitoring practice. Our research group recently reported in-depth on the methodology, showing that low-dose ICG can be used successfully to monitor baseline organ perfusion [15].

Our study has several strengths and limitations. Its strength lies in its standardized design, which followed a fixed protocol in all animals and organs, making the resulting data comparable and reproducible. It was conducted by a multidisciplinary team of medically trained surgeons and veterinary specialists and followed the ARRIVE guidelines for animal research.

Regarding limitations, we did only receive a sufficient quantification signal in 2 of the 3 spleens. This is most likely explained by insufficient priming with ICG at the beginning of the surgery. The dosage given to each study subject was weight-adjusted and did not consider that different organs might need different ICG quantities to provide a strong enough signal for quantitative perfusion assessment. While these results are suboptimal, they add new information to our knowledge of how ICG works in the bloodstream, addressing that different organs have an individual need for what we consider the minimum ICG dosage to achieve a detectable perfusion signal.

In future studies, each subject should receive an individual priming dose to facilitate usable baseline measurements in every organ. We used the Stryker 1588 platform, which necessitated switching between white light during surgery and “ICG-view” during the perfusion recording. This caused a delay in quantitative perfusion assessment after arterial occlusion and necessitated placing “new” ROIs each time the camera was switched to NIR light. While we aimed to select the same ROIs in every measurement, ROI selection varied slightly between conditions due to “white light disruption.” Ideally, a surgical platform with an integrated “overlay” function (or “split-screen”) should be used in future trials to allow the surgeon to operate while simultaneously running the perfusion assessment on the same camera image. Lastly, the camera had to be placed stationary, without movement, to ensure a usable perfusion recording. Movements of the camera result in futile measurements that cannot be used for quantitative analysis. The software mitigates small movements but needs to be developed further before being ready for clinical use as a “background surveillance” of hypoperfused tissue.

Low-dose, high-frequency ICG perfusion monitoring presents a new method for intraoperatively measuring and detecting target-specific hypoperfusion. Further research is needed to validate and improve the method until it can offer a substantial help to surgeons.

The methodology needs to be validated in humans. Based on our findings and since ICG is already widely used during surgical procedures and the cumulative ICG dosage administered stays below the max. recommended dosage, clinical testing is possible and should be the next step in development [22].

Technological improvements should be directed toward the software running “quietly” on the laparoscopic camera screen, continuously deploying a background analysis, and notifying the surgeon in real time when hypoperfusion is detected.

In conclusion, we found that continuous organ-perfusion monitoring using a high-frequency, low-dose ICG bolus regimen can detect organ hypoperfusion in real time. Further validation and development are needed.
